# Gastric Hematamesis, Nicarious of Regular Menstruation, Produced by Malarial Causes

**Published:** 1873-09

**Authors:** E. M. Vasser

**Affiliations:** Alabama


### GASTRIC HEMATAMESIS, VICARIOUS OF REGULAR MENSTRUATION, PRODUCED BY MALARIAL CAUSES.

BY E. M. VASSER, M.D., OF ALABAMA.

*Gentlemen of the Association:*

Though not a member of your body, I take pleasure in reporting a case under the above caption, which is the first of the kind that has come under my observation during a practice of twenty-two years. Though some of you may have met with *one* or more produced by the same influence, yet it may not prove uninteresting to discuss it during your session.

The subject of this is a white woman aged thirty-nine, the mother of several children. Her general health had been good until the close of the war. Her husband, who was a member of Captain Lewis' company of cavalry, was supposed to have been killed or taken prisoner in an attack upon the Federals at the court-house at LaFayette, near Rome, Ga. Not being positive as to the fate that had befallen him, she looked anxiously, but in vain, his coming from some northern bastile, until all hope was gone. The troubled mental condition so impaired the physical, that she found herself unable to labor for the support of herself and children, and was admitted into the poor-house in the early part of 1866, where she soon after had an attack of meningitis. This institution is situated in the fork of the Cahaba and Alabama rivers, immediately on the banks of the former, with a ravine in its rear connecting the two, with ponds and marshes near enough to style its locality the nursery of malaria. Soon after her recovery from meningitis, she was attacked with intermittent fever of the quotidian type. Up to this time, her menstrual flow had occurred at the usual time, in the usual quantity and duration. She saw nothing more, however, for twelve months. Epistaxis occurring occasionally, the spleen became enlarged, with the rise of fever following the first chill, was sore to the touch, and continued to enlarge until she had the appearance of being enciente; and near the time of parturition, the bowels were generally loose. A feeling of vertigo came on all at once, with nausea attending, and hematamesis followed. The organ decreased in size, apparently, all at once, and a short time after, the menstrual flow came on in its natural way, and continued for three successive times; then another chill, and another, for some months, and another hemorrhage from the stomach and bowels—from the stomach several times in the course of half an hour, and by the bowels for several days after, in clots having the appearance of dark, grumous blood. I am told by the patient that she is aware, always, of an attack before it comes on; she says her spleen becomes so much enlarged that it fills the whole abdomen, and that she is nauseated, giddy-headed, and that it is painful for her to stoop or walk about; she therefore goes to bed and awaits the hemorrhage, which never occurs when she is not having chills and the spleen not enlarged, and she always menstruates in the natural way when the spleen is of the natural size, and is free from the ague. She tells me that the hemorrhage has been so great as to cause persons around her to feel alarmed for her safety. These hemorrhages occur at different intervals—as often as five times in one year; owing, in every instance, however, to the size of the spleen—if I may so express it. She has been under the treatment of gentlemen of enlarged experience—one of them my former partner, Dr. Thomas Hunter, with whom I have often conversed respecting her case and treatment. So fully convinced was her physician in attendance several years ago, that a change would be beneficial to her. He advised her to go to the country and remain a few months. She did so, and the result was, the chills were broken by the remedies that were prescribed her, her menstrual flow came on at regular intervals, her general health improved, and she returned to the poor-house in the latter part of June, and in the month of July was taken with a chill, to pass through the same ordeal as related before. I was called to see her in January last, and she had just had one of these hemorrhages. Since then, occasional epistaxis, with a tendency to ascites and diarrhœa; and though she has had a chill or two since, she says the spleen is not sufficiently enlarged for another one of those hemorrhages. As regards the treatment, it has been so varied and of such long duration, I will not attempt a report of it. As regards the prognosis, I would say, in my humble opinion, favorable, if she could leave the poor-house and breathe pure air, not impregnated with malaria; but to remain where she is, then yon can base your own conclusions. "We are told by different authors that the viscera with the morbid conditions of which hematamesis is most often connected, are the liver and spleen, and that disease of one or both of these viscera may produce mechanical congestion of the sub-mucous capillary tissues, and that congestion may be relieved under certain circumstances by the effusion of serous fluid constituting ascites or diarrhœa; or, on the other hand, by extravasation of the collected blood itself. One of the offices of the spleen is supposed to provide a receptacle or reservior of the blood, when its free passage through the portal vessels are temporarily obstructed, and it then becomes a safety-valve which obviates the danger that might arise to more vital parts from any great or sudden disturbance of the venous circulation. Many cases of hematamesis are reported, going on with evident enlargement of the spleen, showing venous obstructure somewhere; but this is the first one that has ever come under my observation where hematamesis is produced, as I believe, by malarial influence, vicarious of menstruation. I have seen the spleen, in this instance, of enormous size, and, from her own description, would weigh, instead of eight or ten ounces, certainly as many pounds, and diminish so rapidly, that one would almost doubt his own eyes.

"Latour, in his work on hemorrhage, mentions a case of a person living in a malarious district who labored under intermittent fever for two years, followed by an immense enlargement of the spleen, or 'ague cake,' which came to occupy almost the whole of the abdomen. He predicted hemetamesis would follow, which accordingly did. He was called to see him, and found that he had vomited an immense quantity of blood, and a great deal passed by the bowels—the hemorrhage recurring from time to time until, in the course of a month, the spleen was so far reduced in size, that it could no longer be felt in the belly, and the patient lived and enjoyed good health for twenty-five years." In gastric hemorrhage, resulting from hepatic trouble or obstruction, there is almost always intestinal hemorrhage.

Finding I am extending my remarks too long, I will close by submitting my statement of the case to my friend, Dr. B. H. Riggs, who will read it before your Association, should he think it worthy of a passing notice, and my feeble health or professional services prevent me from being present at your next meeting.

*Cahaba, Ala., May* 7, 1873.

